# Management of extensively drug-resistant *Acinetobacter baumannii* bacteremia with cefiderocol and sulbactam-durlobactam: a case report

**DOI:** 10.1128/asmcr.00069-24

**Published:** 2025-05-30

**Authors:** E. J. Kim, M. Snyder, K. Macaluso, A. Sonyey, F. Segovia, D. Finkel, D. Cennimo, E. L. Kim

**Affiliations:** 1 Department of Medicine, Rutgers New Jersey Medical School 12286, Newark, New Jersey, USA; 2 United States Department of Veterans Affairs, East Orange, New Jersey, USA; 3 Division of Infectious Disease, Department of Medicine, Rutgers New Jersey Medical School, Newark, New Jersey, USA; 4 Division of Infectious Disease, Department of Medicine, United States Department of Veterans Affairs, East Orange, New Jersey, USA; Rush University Medical Center, Chicago, Illinois, USA

**Keywords:** *Acinetobacter baumannii*, carbapenem-resistance, sulbactam-durlobactam, cefiderocol

## Abstract

**Background:**

Extensively drug-resistant (XDR) *Acinetobacter baumannii* presents an urgent threat to public health due to its significant morbidity and mortality, with limited evidence-based treatment options. Recent drug developments of cefiderocol (FDC) and sulbactam-durlobactam (SUD) provide novel treatment options for XDR *A. baumannii*.

**Case Summary:**

We present the case of a patient with XDR *A. baumannii* bacteremia following a prolonged hospital course for gangrenous cholecystitis and hospital-acquired bacterial pneumonia. He completed a 14-day course of FDC and SUD with clinical resolution and was discharged home.

**Conclusion:**

This case demonstrates the potential efficacy of FDC combined with SUD in treating XDR *A. baumannii* bacteremia, highlighting the need for further research into combination therapy regimens for XDR *A. baumannii*.

## INTRODUCTION

Extensively drug-resistant (XDR) *Acinetobacter baumannii* poses a significant nosocomial threat, with salvage regimens associated with 45% mortality and prolonged hospital stays. Early identification and prompt, effective treatment are critical (1, 2). Cefiderocol (FDC) is a siderophore cephalosporin that utilizes the iron transport system to penetrate gram‐negative bacilli and shows *in vitro* activity against multidrug resistant (MDR) Enterobacterales; however, its monotherapy efficacy against XDR *A. baumannii* remains controversial (3–5). Sulbactam-durlobactam (SUD) combines sulbactam with a novel β‐lactamase inhibitor to overcome diverse *Acinetobacter* resistance mechanisms. Despite FDA approval for hospital-acquired bacterial pneumonia (HABP) and demonstrated reductions in all-cause mortality, SUD’s use in bacteremia remains limited (6). Thus, combination therapy is employed to ensure at least one active agent. Randomized clinical trials treating XDR *A. baumannii* are limited and vary in antibiotic regimens. We present the case of XDR *A. baumannii* bacteremia in a critically ill patient treated with a 14-day course of SUD and FDC, resulting in clinical resolution of bacteremia.

## CASE PRESENTATION

A 61-year-old male was transferred from an outside facility with suspected HABP after an open cholecystectomy for gangrenous cholecystitis 5 days earlier. Two days before transfer, his chest x-ray revealed bilateral infiltrates, and he started on a 7-day course of piperacillin-tazobactam (TZP). Upon transfer, TZP was continued.

On hospital day (HD) 7, he developed worsening respiratory failure requiring intubation. TZP was continued, and intravenous (IV) vancomycin (VAN) was added for the empiric HABP/sepsis protocol. On HD 8, he became hypotensive—requiring two vasopressors—and acute kidney injury (AKI) requiring hemodialysis. On HD 9, antimicrobial coverage was broadened to meropenem and micafungin due to clinical deterioration, and VAN was switched to linezolid for possible vancomycin-resistant enterococci (VRE). Bedside ultrasound showed fluid at the prior gallbladder fossa, but further imaging was not obtained due to his critical condition. By HD 13, the patient became afebrile with multiple sputum, bronchoalveolar lavage, and blood cultures (cx) negative for any growth. Computerized tomography (CT) scan of the abdomen and pelvis (CT AP) obtained after clinical improvement did not show fluid collection at the recent surgical site. The decision to stop all antimicrobials was made on HD 17. Vasopressors were discontinued on HD 19.

On HD 21, worsening leukocytosis and tachycardia prompted blood, urine, and sputum cultures. Sputum cx grew MDR *Providencia stuartii*. With clinical improvement after volume removal and prior to culture results, antibiotics were held. Prolonged intubation with multiple failed spontaneous breathing trials necessitated a tracheostomy on HD 24. On HD 32, he became febrile (100.4^o^F) with tachycardia to 100–120 s bpm (beats per minute), and sputum cx grew *Klebsiella pneumoniae*, phenotypic XDR *A. baumannii*, and MDR *P. stuartii* (see Table 1). He was monitored off antibiotics as fever self-resolved and respiratory status remained stable. The patient’s labs and vitals continued to improve with resolution of AKI and was downgraded to the medical floor on HD 34.

**TABLE 1:** Culture and sensitivity reports^*a*^

Antibiotic	Interpretation (MIC μg/mL)					
	Sputum culture			Blood culture		
	HD 32			HD 37	HD 38	
	*Klebsiella* *pneumoniae*	*Acinetobacter* *baumannii*	*Providencia* *stuartii*	*Klebsiella pneumoniae*	*Acinetobacter baumannii*	*Providencia* *stuartii*
Amikacin	*S* (≤2)	***R***	*S* (≤2)	*S* (≤2)	***R***	*S* (≤2)
Gentamicin	*S* (≤1)	***R* (≥16**)	*S* (2)	*S* (≤1)	***R* (≥16**)	*S* (4)
Tobramycin	*S* (≤1)	***R* (≥16**)	*S* (2)	*S* (≤1)	***R* (≥16**)	*S* (2)
Ampicillin	***R* (≥32**)	***R***	***R* (≥32**)	***R* (≥32**)	***R***	***R* (≥32**)
Ampicillin/sulbactam (SAM)	—^*b*^	***R* (16**)	—	—	***R* (≥32**)	—
TZP	*S* (8)	***R* (≥128**)	*I* (32)	*S* (8)	***R* (≥128**)	*S* (8)
Cefazolin	*S* (≤4)	—	***R* (≥64**)	*S* (≤4)	***R* (≥64**)	***R* (≥64**)
Cefoxitin	*S* (≤4)	—	***R* (≥64**)	*S* (≤4)	—	***R* (≥64**)
Ceftizoxime	*S* (≤1)	—	***R* (≥64**)	*S* (≤1)	—	***R* (≥64**)
Cefepime	*S* (≤1)	***R* (≥64**)	*S* (2)	*S* (≤1)	***R* (≥64**)	*S* (4)
Cefiderocol	*S* (0.12)	***R* (8**)	—	—	—	—
Ertapenem	*S* (≤0.5)	—	***R* (≥8**)	*S* (≤0.5)	—	***R* (≥8**)
Imipenem	*S* (0.5)	***R* (≥16**)	—	*S* (0.5)	***R* (≥16**)	*S* (4)
Meropenem	*S* (≤0.25)	***R* (≥16**)	*S* (4)	*S* (≤0.25)	***R* (≥16**)	*S* (4)
Ciprofloxacin	*S* (≤0.25)	***R* (≥4**)	***R* (≥4**)	*S* (≤0.25)	***R* (≥4**)	***R* (≥4**)
Levofloxacin	*S* (≤0.12)	***R* (≥8**)	*I* (4)	*S* (≤0.12)	***R* (≥8**)	*I* (4)
Tetracycline	*S* (2)	***R* (≥16**)	***R* (≥16**)	*S* (2)	***R* (≥16**)	***R* (≥16**)
Tigecycline	*S* (1)	*I* (4)	—	*S* (1)	*I* (4)	*S* (2)
Minocycline	*S* (2)	*S* (2.0)	—	—	—	—
Trimethoprim-sulfamethoxazole (TRM-SULF)	*S* (≤2/38)	***R* (≥4/76**)	*S* (≤2/38)	*S* (≤2/38)	***R* (≥4/76**)	*S* (≤2/38)
Colistin	*S* (≤0.25)	*S* (0.5)	—	—	—	—

^*a*^ Identification and susceptibility were determined using BioMérieux’s Vitek 2 (GN69 and XN06 panels) and tested per institution policy. FDA breakpoint cutoffs were used for all susceptibility testing as per institution policy. Boldface type is used to highlight resistance.

^*b*^ — indicates testing was not done due to known intrinsic resistance, irrelevance of that antibiotic-organism combination, or adherence to established clinical guidelines.

On HD 37, the patient became febrile again (101.2°F) and developed leukocytosis. Blood cx via BioFire Blood Cx Identification Panel (BCID) was positive for *K. pneumoniae* without resistance genes detected. Extended infusion TZP (3.375 g every 8 hours over a 3-hour infusion) was started. On HD 38, BioFire BCID detected *A. baumannii* without IMP, VIM, NDM-1, KPC, OXA-48, or mcr-1 resistance genes. Identification and susceptibility using BioMérieux’s Vitek 2 (GN69 and XN06 panels) confirmed phenotypic-XDR *A. baumannii* and phenotypic-MDR *P. stuartii* (see Table 1).

**Fig 1:**
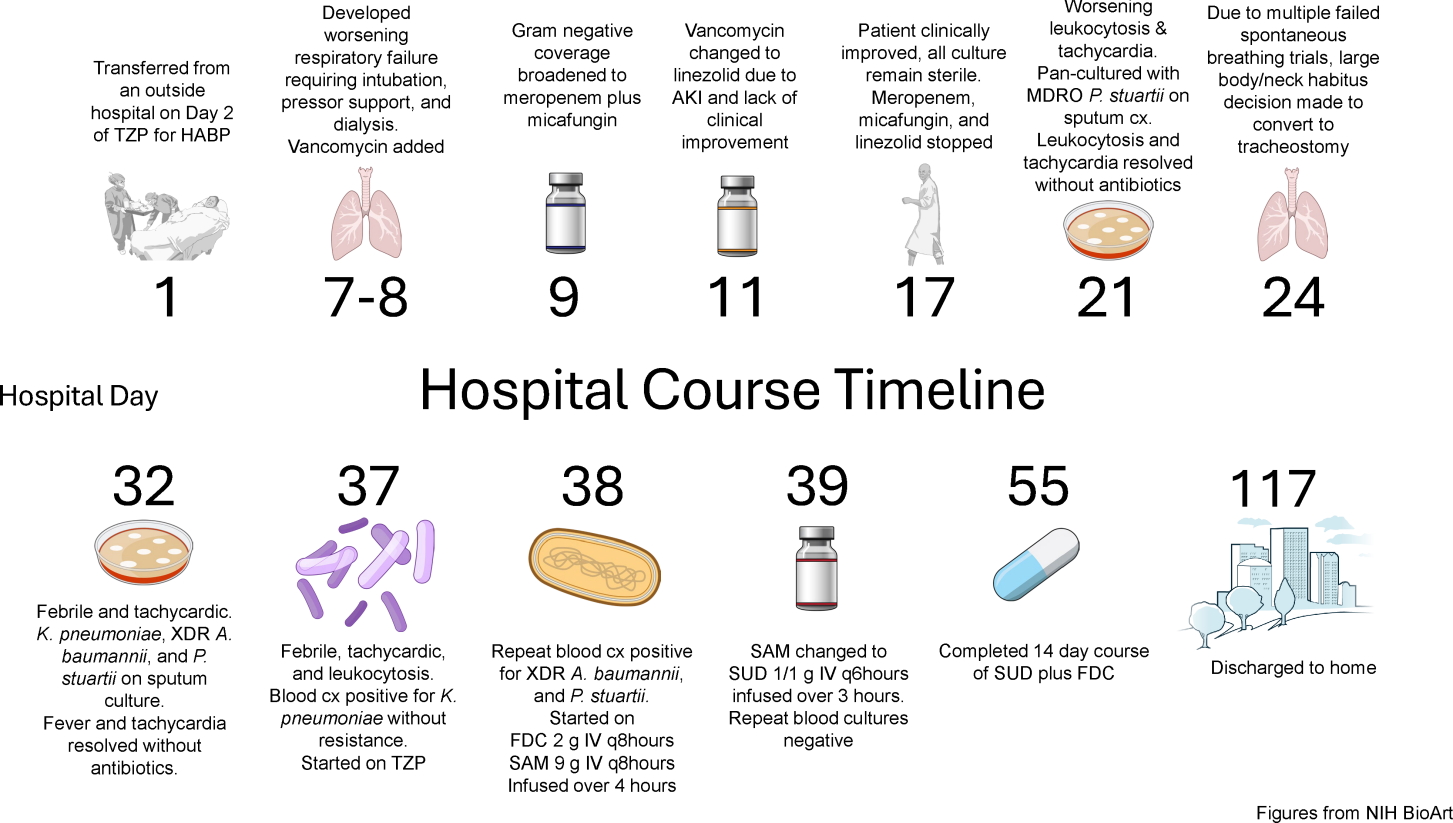
Hospital course timeline.

Based on prior sputum cx (see Table 1) results and limited data on the treatment for XDR *A. baumannii* bacteremia, the following regimen was started on HD 38: FDC 2 g IV every 8 hours (q8h) administered as a 3-hour infusion, plus high-dose ampicillin-sulbactam (SAM) 9 g IV q8h, given as a 4-hour infusion, with plans to switch SAM to SUD. On HD 39, repeat blood cultures were cleared, and SUD was procured the same day. After confirming Y-site compatibility with FDC, SAM was replaced with SUD (1 gm/1 gm IV q6h) and administered as a 3-hour infusion (1). The patient’s fevers, tachycardia, and leukocytosis were resolved. He completed a 14-day total course of FDC and SUD. Minocycline, colistin, and FDC susceptibility were unable to be performed in-house and were outsourced on HD 38. On HD 95, susceptibility results became available (see Table 1). Notably, *A. baumannii* was FDC-resistant based on FDA breakpoints (7). Due to logistical issues, SUD susceptibility could not be confirmed. The patient was able to be discharged home on HD 117. At the 6-month follow-up, the patient Fig. 1 had no recurrence of bacteremia. A summary of the hospital course can be found in Fig. 1.

## DISCUSSION

Several degrees of drug resistance were standardized by a joint initiative between the Centers for Disease Control and Prevention and the European Centre for Disease Prevention and Control. These include MDR, defined as resistance to at least one agent in three or more antimicrobial categories; XDR, resistance to at least one agent in all but two or fewer antimicrobial categories; pan drug-resistance, indicating resistance to all agents in all antimicrobial categories (8). Organisms such as XDR *A. baumannii* are often associated with severe infections with high mortality rates, prompting the development of novel treatment options, including FDC and the recently FDA-approved SUD (1, 9).

FDC is a catechol-type siderophore cephalosporin that utilizes the microbial siderophore-iron pathway to penetrate gram-negative organisms, allowing the cephalosporin to enter the bacterial periplasmic space and bind to the penicillin-binding proteins (PBP) (10, 11). Initially approved for complicated urinary tract infections, *in vitro* data indicated potent activity against gram-negative pathogens, including XDR *A. baumannii* (12). However, FDC as a monotherapy remains controversial (3, 4, 13, 14).

In the *Acinetobacter*, *Pseudomonas*, *Escherichia coli*, *Klebsiella*, *Stenotrophomonas* - nosocomial pneumonia (APEKS-NP) (NCT03032380) trial of 251 patients with severe gram-negative nosocomial pneumonia (16 were XDR *A. baumannii* infections), FDC was non-inferior in all-cause mortality on day 14 compared to high-dose, extended infusion of meropenem (12.4% vs 11.6%) (4). A Multicenter, Randomized, Open-label Clinical Study of S-649266 or Best Available Therapy for the Treatment of Severe Infections Caused by Carbapenem-Resistant Gram-negative Pathogens (CREDIBLE-CR) (NCT02714595) trial also found similar efficacy of FDC combination therapy compared to best available therapy (BAT) for carbapenem-resistant gram-negative bacteria. Alarmingly, the FDC-monotherapy group increased in all-cause mortality compared to BAT (49% vs 18%). Post-hoc analysis suggests the increase in FDC monotherapy mortality was driven by the patients with XDR *A. baumannii* pneumonia and bloodstream infections (15).

Since FDA approval, FDC resistance has been reported (14, 16). A meta-analysis and systematic review by Karaokonstantis et al. found that 8.8% of over 6,000 *Acinetobacter* isolates were resistant to FDC, with mechanisms including β-lactamases (NDM, KPC, AmpC, OXA-427, and PER- and SHV-type ESBLs), porin mutations, siderophore receptor mutations, efflux pump activity, and modifications to PBP3. Additionally, the authors observed that resistance could be conferred through multiple mutations, with heteroresistance highly prevalent in these isolates (5). Notably, FDC resistance was noted in our patient.

SUD is a combination β-lactam-β-lactamase inhibitor (BLI) primarily developed for treating drug-resistant *A. baumannii*. Sulbactam, a β-lactam antibacterial, targets a subset of Ambler class A enzymes that can inhibit bacterial cell wall synthesis by binding to PBP. Alone, sulbactam is susceptible to degradation, with class D β-lactamases being the primary β-lactamase for sulbactam degradation. Durlobactam, which belongs to the family of diazabicyclooctanone BLI, inhibits Ambler classes A, C, and D β-lactamases and is thus critical to SUD’s efficacy against XDR *A. baumannii* (17, 18). The FDA approved SUD on May 23, 2023, for hospital-acquired and ventilator-associated bacterial pneumonias. In the ATTACK trial, a phase III multicenter study, SUD demonstrated a decrease in 28-day all-cause mortality amongst XDR *A. baumannii* infections treated with SUD and imipenem-cilastatin vs those treated with colistin and imipenem-cilastatin (19% vs 32%). Only five patients of the 181 patients in the randomized group had XDR *A. baumannii* bacteremia (6).

Resistance to SUD among *Acinetobacter* species has also been reported in the literature. 2.3% of isolates exhibited resistance, with prevalence increasing to 3.4% among XDR *A. baumannii* isolates and 3.7% among colistin-resistant strains (19). The primary mechanisms underlying SUD resistance include the production of metallo-β-lactamases, such as NDM-1, and mutations in PBP3 (19–21). In our case, the genotypic resistance mechanism was not identified; however, we did show phenotypic resistance with BioMérieux’s Vitek 2 (GN69 and XN06 panels). This case highlights the utility of testing molecular resistance markers beyond the BioFire BCID2 panel in dangerous organisms such as XDR *A. baumannii*, as the BioFire BCID2 panel may not identify the carbapenem resistance gene found in *A. baumannii*.

Most literature addresses XDR *A. baumannii* infections treated with colistin monotherapy vs together with other drug combinations. Apart from the ATTACK trial, we found only one case report of XDR *A. baumannii* HABP treated successfully with FDC and SUD, and only one case report of XDR *A. baumannii* HABP treated successfully with SUD and meropenem (22, 23). To our knowledge, this is the first case report documenting the successful treatment of phenotypic XDR *A. baumannii* bacteremia with a combination of FDC + SUD. Further investigation on the safety and effectiveness of FDC and SUD—alone or in combination—could help guide and optimize treatment against XDR *A. baumannii*.

This study has several limitations. First, no microbiology results were available for the initial gangrenous cholecystitis because cultures were not obtained by the outside facility. Second, only the phenotypic resistance of XDR *A. baumannii* was demonstrated, as genotypic resistance testing beyond the BioFire BCID2 panel was not performed. This case highlights the utility of testing molecular resistance markers beyond the BioFire BCID2 panel in dangerous organisms such as XDR *A. baumannii*. Third, susceptibility testing for SUD was unavailable due to logistical issues.

## Data Availability

Data sharing is not applicable to this article; no new data sets were generated or analyzed during the current study.
